# Personalized Risk Analysis to Improve the Psychological Resilience of Women Undergoing Treatment for Breast Cancer: Development of a Machine Learning–Driven Clinical Decision Support Tool

**DOI:** 10.2196/43838

**Published:** 2023-06-12

**Authors:** Georgios C Manikis, Nicholas J Simos, Konstantina Kourou, Haridimos Kondylakis, Paula Poikonen-Saksela, Ketti Mazzocco, Ruth Pat-Horenczyk, Berta Sousa, Albino J Oliveira-Maia, Johanna Mattson, Ilan Roziner, Chiara Marzorati, Kostas Marias, Mikko Nuutinen, Evangelos Karademas, Dimitrios Fotiadis

**Affiliations:** 1 Foundation for Research and Technology-Hellas, Institute of Computer Science Heraklion Greece; 2 Department of Materials Science and Engineering, University of Ioannina Ioannina Greece; 3 Foundation for Research and Technology-Hellas, Biomedical Research Institute Ioannina Greece; 4 Comprehensive Cancer Center, Helsinki University Hospital University of Helsinki Helsinki Finland; 5 Applied Research Division for Cognitive and Psychological Science, European Institute of Oncology Istituto di Ricovero e Cura a Carattere Scientifico Milan Italy; 6 Department of Oncology and Hemato-Oncology University of Milan Milan Italy; 7 School of Social Work and Social Welfare,The Hebrew University of Jerusalem Jerusalem Israel; 8 Breast Unit, Champalimaud Research and Clinical Centre, Champalimaud Foundation Lisbon Portugal; 9 Champalimaud Research and Clinical Centre, Champalimaud Foundation Lisbon Portugal; 10 Department of Communication Disorders, Sackler Faculty of Medicine, Tel Aviv University Tel Aviv Israel; 11 Nordic Healthcare Group Helsinkifin Finland

**Keywords:** breast cancer, classification, machine learning, mental health, well-being, explainability, interventions, risk assessment

## Abstract

**Background:**

Health professionals are often faced with the need to identify women at risk of manifesting poor psychological resilience following the diagnosis and treatment of breast cancer. Machine learning algorithms are increasingly used to support clinical decision support (CDS) tools in helping health professionals identify women who are at risk of adverse well-being outcomes and plan customized psychological interventions for women at risk. Clinical flexibility, cross-validated performance accuracy, and model explainability permitting person-specific identification of risk factors are highly desirable features of such tools.

**Objective:**

This study aimed to develop and cross-validate machine learning models designed to identify breast cancer survivors at risk of poor overall mental health and global quality of life and identify potential targets of personalized psychological interventions according to an extensive set of clinical recommendations.

**Methods:**

A set of 12 alternative models was developed to improve the clinical flexibility of the CDS tool. All models were validated using longitudinal data from a prospective, multicenter clinical pilot at 5 major oncology centers in 4 countries (Italy, Finland, Israel, and Portugal; the Predicting Effective Adaptation to Breast Cancer to Help Women to BOUNCE Back [BOUNCE] project). A total of 706 patients with highly treatable breast cancer were enrolled shortly after diagnosis and before the onset of oncological treatments and were followed up for 18 months. An extensive set of demographic, lifestyle, clinical, psychological, and biological variables measured within 3 months after enrollment served as predictors. Rigorous feature selection isolated key psychological resilience outcomes that could be incorporated into future clinical practice.

**Results:**

Balanced random forest classifiers were successful at predicting well-being outcomes, with accuracies ranging between 78% and 82% (for 12-month end points after diagnosis) and between 74% and 83% (for 18-month end points after diagnosis). Explainability and interpretability analyses built on the best-performing models were used to identify potentially modifiable psychological and lifestyle characteristics that, if addressed systematically in the context of personalized psychological interventions, would be most likely to promote resilience for a given patient.

**Conclusions:**

Our results highlight the clinical utility of the BOUNCE modeling approach by focusing on resilience predictors that can be readily available to practicing clinicians at major oncology centers. The BOUNCE CDS tool paves the way for personalized risk assessment methods to identify patients at high risk of adverse well-being outcomes and direct valuable resources toward those most in need of specialized psychological interventions.

## Introduction

### Background

Coping with breast cancer (BC) is increasingly becoming a major socioeconomic challenge in high-, middle-, and low-income countries worldwide. This is due to the constantly rising incidence of the disease along with rapidly declining mortality rates [1]. Although increasingly treatable, BC and related medical treatments remain a very stressful and potentially life-changing experience for many women [2]. The process of successful adaptation to illness can be conceptually characterized as a person’s *psychological resilience*. Adequate resilience is typically indicated by the ability to maintain good emotional functioning in the face of illness (eg, maintaining low levels of psychological symptoms) or return to normal function shortly after adversity [3]. Thus, health professionals are often faced with the need to identify women at risk of manifesting poor resilience to the illness and even more so to identify risk factors of poor resilience for a given patient. The latter step is paramount for designing personalized psychological interventions to promote well-being in women with BC. This paper describes the development and implementation of an IT solution in the form of a clinical decision support (CDS) tool to address this need. This CDS tool was the product of a multicenter, multinational project entitled “Predicting Effective Adaptation to Breast Cancer to Help Women to BOUNCE Back” (BOUNCE).

### Is Psychological Resilience Predictable?

Extensive research in the field of psychology over the past decades has identified several types of patient characteristics, including biological, clinical, sociodemographic, and lifestyle variables, that can account for and predict levels of resilience [4,5]. These models have informed clinical practice by highlighting a wide range of coping skills, behaviors, and emotion regulation strategies to serve as targets in psychological prevention and intervention programs to help women bounce back from BC and resume normal lives. However, the conclusions of previous studies are not very practical in routine clinical practice for 2 main reasons. First, the extent of the psychological evaluation required to cover all potentially relevant aspects of resilience (typically in the form of several, often extensive self-report questionnaires [6]) is vast, especially if more than one measurement point is involved. Second, even if such an extensive evaluation reveals the presence of a high risk of poor resilience for a given patient, conventional statistical models are not capable of specifying the most crucial individual risk factors that would permit the design of a personalized prevention or intervention program. Therefore, more work is needed to identify better predictive markers as well as comprehensive models that can be applied in clinical practice.

### Advantages and Challenges of IT Solutions

Machine learning (ML) models are inherently suitable for addressing the challenges posed by multimodal data sets such as those explored in previous studies of resilience, including (1) scalability, (2) high dimensionality, (3) heterogeneity and complexity, and (4) distribution of the data [7,8]. A key advantage of these methods is their ability to automate the process of hypothesis generation and evaluation in comparison with conventional statistical approaches. Their inherent ability to assign parameter weights based on correlations with the binary outcome makes this type of analysis an appealing choice considering the growing volume and complexity of multimodal, multi-scale data. These approaches can further serve to reduce the number of potential predictors examined in new research and the extent of assessment required, thus reducing the burden for patients.

ML and artificial intelligence (AI) are gradually becoming crucial tools in various areas of health care research. Nevertheless, explainability; transparency; and, most importantly, accountability and responsibility do not receive due consideration, in part because it remains difficult to transform these necessary concepts (eg, trust) into actual computational tools or metrics. Understanding why and how a particular model produced the observed predictions is of paramount importance, especially in health care applications. Without investigating the models in more depth, there is always the possibility of erroneous results, overfitting, or fitting using spurious and unimportant features and characteristics. *Explainable AI* (XAI) is the general term for a wide array of computational tools designed to improve the understanding of the underlying mechanisms driving the results of ML-based predictions [9-12]. Apart from validating a model’s results and performance, XAI can also be used to explore individualized differences at the patient level and produce personalized risk profiles and interpretable ML-based models. Several studies have used well-established ML algorithms for modeling BC progression and survival to identify predictors of distinct disease outcomes [13-15]. Common tasks involve the prediction of cancer susceptibility, cancer recurrence or local control, and cancer survival.

However, to date, adaptive learning algorithms have been sparsely used to address end points related to the psychological well-being of women throughout the critical 12- to 18-month period of cancer treatments and physical recovery. Certain sociodemographic, lifestyle, and psychological characteristics are suspected determinants of patients’ subsequent overall quality of life (QoL) and mental health. To the best of our knowledge, little to no work has focused on providing clinicians with personalized predictions and clinical recommendations through explainable and transparent AI-based CDS tools. An extensive literature survey, undertaken between 2016 and 2021, found only 4 out of 46 articles with mental health as their primary study and AI as the core methodology behind their proposed CDS tools. Although the review underlined the importance of developing and delivering such applications in clinical practice, the road to translating AI in mental health care delivery was reported to be still obscured by lack of trust and poor interpretability [16]. In addition, the eligible studies for this review were limited because of their small sample size, the hypothesis behind the examined patient scenarios, and the absence of personalized recommendations. A recent ML-driven CDS tool using physical, psychiatric, and social factors to predict QoL in older adults demonstrated adequate performance without, however, linking model results to clinical recommendations or assessing model uncertainty and explainability [17].

### The Rationale of the BOUNCE Proposed Solution

To address this need, an interdisciplinary consortium of experts was formed in 2017 in response to a Horizon 2020 call for personalized medicine research and innovation solutions. The overreaching goal of BOUNCE [18] was to examine how women adapt to BC. At the core of the project was a multicenter clinical pilot at 5 major oncology centers in 4 countries (Italy, Finland, Israel, and Portugal) enrolling 706 patients with highly treatable BC, most of whom were followed up for 18 months. The comprehensive data set compiled through BOUNCE included psychological, demographic, lifestyle, clinical, and biological characteristics that were recorded shortly after diagnosis and before experiencing any side effects from systemic therapies. In the context of BOUNCE, resilience was defined by three complementary patient-reported well-being outcomes: (1) absence of considerable anxiety or depression symptomatology, (2) adequate global QoL, and (3) maintenance of low levels of anxiety or depression symptoms through BC treatments and recovery. Specifically, BOUNCE planned to develop a computational tool to (1) identify women at risk of poor well-being outcomes following a diagnosis of BC; (2) specify psychological, demographic, lifestyle, clinical, and biological characteristics that contribute most to the successful recovery of women with BC in general; (3) identify potentially modifiable psychological and lifestyle characteristics that, if addressed systematically in the context of personalized psychological interventions, are most likely to promote resilience in a given patient; and (4) use robust feature selection techniques to improve the clinical feasibility of the required psychological assessments given the vast number of potentially relevant predictors highlighted in the extant literature.

### Design of the BOUNCE CDS Tool

Clinicians who are called in to support women likely to show poor illness adaptation are often challenged with the task of choosing therapeutic targets that can be directly and efficiently pursued in the context of relatively brief psychological interventions. To meet these clinical needs, the BOUNCE CDS tool was designed according to four principles: (1) flexibility for future use in clinical settings, (2) performance accuracy in predicting key aspects of patient well-being, (3) robustness in formulating personalized risk profiles of potentially modifiable patient characteristics (model explainability), and (4) directly linking personalized needs assessment with concrete suggestions regarding psychological prevention or intervention strategies.

*Flexibility* means that clinicians can adapt the CDS tool to their clinical needs for a given patient. Accordingly, we developed models capable of assessing the risk of 6 distinct well-being outcomes (overall mental health, mental health decline, and global QoL decline over a 12- or 18-month period). Additional clinical flexibility is afforded in terms of the available data in mainstream clinical practice (ie, clinical, psychological, and lifestyle or clinical/biological measurements before BC treatment onset and at 3 or 6 months during the course of the illness). The *performance* principle means that the selected models were those that displayed adequate classification accuracy through extensive cross-validation schemes on the BOUNCE prospective clinical study data set. Moreover, *model explainability* in terms of individual characteristics placing a given patient at risk of adverse well-being outcomes relied on model-agnostic algorithms at the local (patient) level. The CDS tool uses these algorithms to formulate personalized risk profiles of potentially modifiable patient characteristics. This is achieved by estimating the contribution of each level of the predictor variable as derived from the respective classification model to a given probability of predicted class membership (ie, “poor” or “good” future outcome). In effect, model-agnostic analysis plots allow users to visualize the amount of change in a given well-being outcome (eg, severity of depression and anxiety symptoms at 12 months after diagnosis) had the patient shown a specific amount of improvement on a modifiable predictor variable (ie, reduction in negative affect). Finally, the selected models for the BOUNCE CDS tool are directly linked to explicit BOUNCE *clinical recommendations* concerning intervention strategies and techniques with demonstrated efficiency in previous research in the fields of clinical and health psychology and psycho-oncology. The BOUNCE clinical recommendations contain a short list of basic cues and solutions that a health care professional can use to encourage the patient to more actively participate in their treatment and enhance their psychological health and QoL.

The goal of this work was 2-fold. First, we aimed to describe the development of several alternative ML models designed to accommodate various clinical scenarios aimed at identifying BC survivors at risk of poor overall mental health or global QoL. The results are presented in detail for models predicting 12-month overall mental health status in the main text (corresponding results for additional 12-month outcomes as well as all 18-month prediction models are presented in Multimedia Appendix 1 [19-31]). Second, we aimed to describe the potential clinical utility of these models as indicated by their respective performance accuracy and their capacity to specify relevant targets for personalized psychological interventions through XAI analyses.

## Methods

### Participants

Participants were enrolled in the study before the start of oncological treatment, which was typically 2 to 5 weeks after surgery. For patients who received neoadjuvant therapy (ie, chemotherapy before surgery), enrollment took place 2 to 5 weeks after the diagnostic biopsy. This coincided with the first measurement wave of the prospective longitudinal study (“baseline” or month 0) to ensure that patients had not experienced any side effects from systemic therapy. The inclusion criteria were as follows: aged 40 to 70 years, a recent diagnosis of histologically confirmed invasive early or locally advanced operable BC, stage-I to stage-III tumor, and receiving surgery and any type of systemic treatment. Exclusion criteria included distant metastases and a history of another malignancy within the last 5 years.

### Ethics Approval

The study was approved by the ethical committee of the European Institute of Oncology (approval R868/18‐IEO916) and the ethical committees of each participating hospital. All participants were recruited by their oncologists and signed a consent form detailing all study procedures, including access to the hospital medical records as a source of information for medical data, as well as all intended data uses. Data were aggregated into a central database that was used for this modeling work after they had been properly deidentified. Participants did not receive any compensation for their time and effort.

### Measures

#### Predictor Variables

##### Sociodemographic

The following variables were recorded at baseline (month 0): age (in years), educational level (categorized as low [0-9 years] and high [>9 years]), relationship status (single or with partner), children (yes or no), employment status (currently employed or not), type of employment (full time, retired, or self-employed vs unemployed, housewife, or part-time employment), and monthly income (very low vs average/high, adjusted for the gross domestic product of the home country of each participant). A total of 2 additional variables were aggregated over the first 3 months after enrollment (month 3): sick leave taken (in days) and considerable life stressors (other than BC) during the first 3 months after diagnosis (categorized as none or single event vs ≥2 events).

##### Lifestyle (at Month 0)

The following lifestyle characteristics were recorded: current smoking, alcohol consumption (no drinking or occasional consumption, defined as ≤2 servings of beer or ≤1 serving of spirits per week; moderate, defined as 3-6 servings of beer or ≤4 servings of spirits per week; and heavy, defined as >6 servings of beer or >4 servings of spirits per week), self-defined diet (Mediterranean or special diet [eg, vegan or lactose-free], undefined), and physical exercise (low [<60 min per week], moderate [60-180 min per week], and heavy [>180 min per week]).

##### Medical (at Month 0)

A number of clinical and biological variables were extracted from the patients’ medical records: Eastern Cooperative Oncology Group performance status; obesity; family history of BC; preexisting chronic physical illness (other than metabolic); psychotropic medications (including sleep medications); preexisting metabolic disease; preexisting anxiety or dysthymia; anemia; menopausal status (premenopausal, perimenopausal, or postmenopausal); serum levels of alanine aminotransferase, creatinine, and bilirubin; and blood cell count (thrombocyte count and proportion of neutrophils over total leukocyte count).

##### BC-Related Variables

BC-related variables included cancer stage (I vs II or III), tumor molecular profile (luminal A, luminal B, triple negative, or human epidermal growth factor receptor 2 [HER2]–enriched), progesterone receptor positivity, estrogen receptor positivity, HER2 positivity, and Ki67 levels (≥25). Treatment-related variables were surgery at month 0, surgery at month 3, onset of chemotherapy at month 0, onset of chemotherapy at month 3, onset of radiotherapy at month 0, onset of radiotherapy at month 3, type of breast surgery (lumpectomy vs mastectomy), type of chemotherapy (adjuvant or neoadjuvant), type of endocrine therapy (letrozole, exemestane, anastrozole, ovarian suppression, or tamoxifen), anti-HER2 therapy, and systematic mental health support through month 3.

##### Psychosocial Characteristics

Psychosocial characteristics were evaluated using standardized questionnaires that had been appropriately adapted and translated into 5 languages of the BOUNCE participants (Italian, Portuguese, Finnish, Hebrew, and Arabic). The following domains were assessed: (1) several personality characteristics, (2) coping and the ability to cope, (3) perceived social support, (4) resilience as a trait, (5) illness perception and related behaviors, (6) global health status/QoL and anxiety and depression symptoms, and (7) patient affect at the time of measurement. A detailed list of available measures can be found in the study by Pettini et al [32] and in Multimedia Appendix 1 [19-31].

#### Outcome Variables

CDS tool users could choose among three distinct endpoints: (1) overall mental health status at month 12 or month 18 (“good” or “poor”), (2) mental health decline between baseline (month 0) and month 12 (or month 18), and (3) decline in global QoL between baseline (month 0) and month 12 (or month 18). Self-reported overall mental health status was indexed by the total score on the 14-item Hospital Anxiety and Depression Scale. Higher scores indicate more frequent psychological symptoms. The clinically validated cutoff score of 16/42 points in a wide range of languages was used to identify patients who reported potentially clinically significant symptoms [33,34]. Global QoL was assessed using the 2 questions from the Global Health Status scale of the European Organisation for Research and Treatment of Cancer QLQ-C30 (Quality of Life Questionnaire–Cancer) [19]. Higher scores indicate better overall QoL. In the absence of a clinically validated cutoff, we used the lower 25th percentile of the total sample distribution of scores at month 0 to identify patients who rated their QoL as relatively poor (corresponding to a score of 75 points).

### Model Design

A total of 12 models were developed, cross-validated, and incorporated into the BOUNCE CDS tool varying on the type of end point examined by each and on the timing of measurement of predictor variables. Three sets of models were included in the CDS tool in terms of well-being end points at 12 or 18 months after enrollment (Table 1): (1) type A models addressed the need to identify patients at risk of overall poor mental health, (2) type B models addressed the need to identify patients at risk of declining mental health, and (3) type C models were designed to identify patients at risk of declining global QoL.

Type A models were more suitable for patients who reported *poor mental health* at the time of enrollment in the study, whereas type B and C models were designed for patients who reported *good mental health* (or QoL, respectively) at that time.

The primary prediction models incorporated into the BOUNCE CDS tool assume that psychological and lifestyle predictor variables are recorded within a few weeks from the time of diagnosis and shortly after the onset of cancer treatments (up to approximately 3 months after diagnosis). Clinical and biological variables are typically available as part of the patient’s medical file at the time of diagnosis. However, it is often the case that a mental health professional is not called in until sometime later, and therefore, data on potential psychological and lifestyle predictors are not available until approximately 6 months after diagnosis. To accommodate the risk assessment needs for such cases, we designed distinct model subtypes according to the timing of the available predictor measurements (at month 0/month 3 and month 6). Table 2 describes the 2 subtypes of models available for each of the 3 main models (A, B, and C), forming a total of 12 models that were fully tested and included in the BOUNCE CDS tool.

**Table 1:** The 3 types of models incorporated into the Predicting Effective Adaptation to Breast Cancer to Help Women to BOUNCE Back clinical decision support tool according to distinct well-being end points.

End point	Status at baseline	Status at month 12 or month 18	Model type
Overall mental health	Good or poor	Good	A
Overall mental health	Good or poor	Poor	A
Change in mental health	Good	Good (stable)	B
Change in mental health	Good	Poor (declining)	B
Change in QoL^a^	Good	Good (stable)	C
Change in QoL	Good	Poor (declining)	C

^a^QoL: quality of life.

**Table 2:** Subtypes of machine learning models incorporated into the Predicting Effective Adaptation to Breast Cancer to Help Women to BOUNCE Back clinical decision support tool according to the timing of predictor measurement.

Predictor variables measured at the time of diagnosis	Predictor variables measured at 3 months after diagnosis	Predictor variables measured at 6 months after diagnosis	Model subtype
Clinical and biological	Clinical	N/A^a^	i
Lifestyle	Lifestyle	N/A	N/A
Psychological	Psychological	N/A	N/A
Clinical and biological	N/A	Clinical	ii
N/A	N/A	Lifestyle	N/A
N/A	N/A	Psychological	N/A

^a^N/A: not applicable.

### Supervised Learning Analysis Pipeline

An ensemble learning supervised feature selection and classification scheme was developed based on the balanced random forest (BRF) classification model, a variation of the frequently used random forest [35] with internal class-balancing capabilities. More details are presented in the following sections.

#### Data Preprocessing and Handling of Missing Data

Initially, raw data were rescaled to 0 mean and unit variance, and ordinal variables were recoded into dummy binary variables. Cases and variables with >10% of missingness were excluded from the final data set. The remaining missing values were replaced with the global mean values.

#### Feature Selection

Feature selection was performed to select variables of the highest importance for each prediction. The selection was conducted inside a nested cross-validation pipeline using a BRF algorithm that assigns weights to the features and ranks them according to their relative importance. The top 20 features were retained according to cumulative feature importance and entered into the final classification models. Embedding the feature selection process into the nested validation loop ensured stable and representative results and a final robust and generalizable ML predictive model.

#### Model Training and Validation

To address the rather common problem of model overfitting or overly optimistic results in ML applications in clinical research, we adopted a nested cross-validation scheme [36-38]. Model overfitting occurs because a model that has less training error (ie, misclassifications on training data) can have poor generalization (expected classification errors on new unseen data) than a model with higher training error. To address this problem, model testing was always performed on unseen cases that were not considered during the training phase and, consequently, did not influence the feature selection process. Specifically, 80% of the data were used for training in a repeated, stratified 5-fold cross-validation scheme.

#### Classification With BRF Algorithm

The ML pipeline was implemented in Python (Python Software Foundation) using the scikit-learn ML library [39]. Class imbalance was addressed by using random undersampling to balance the subsets combined inside an ensemble. The BRF classifier [40,41] combines the down-sampling majority-class technique and the ensemble learning approach, artificially adjusting the class distribution so that classes are represented equally in each tree in the forest. In this manner, each bootstrap sample contains balanced, down-sampled data. Model performance was evaluated on the following metrics: specificity (true negative rate), sensitivity (true positive rate), accuracy, precision, *F*_1_-score, and area under the curve. All model hyperparameters for both the selection and classification BRF models were left to default values (instead of hyperparameter grid-type search) to enhance the generalizability of the results.

#### ML Explainability Analyses

Model-agnostic analyses were applied to the final cross-validated models, which were trained using the most important 20 features (data from all available participants). Global-level analyses explored the relative importance of the selected variables in distinguishing between the 2 classes and, thus, making accurate 12- or 18-month predictions based on the selected features. Analyses at the local (patient) level searched for key contributors to a given classification result after controlling for all other predictors in the model. This was made possible with the use of ceteris paribus profiles [42-44] as well as breakdown plots [45], both created for individual participants while using the model trained on the remaining participants. The former illustrates the estimated change in prediction (as a continuous metric of class membership) at different levels for each predictor variable. Ceteris paribus profiles and breakdown plots were developed using the *dalex* Python package [46,47] with the default values in the arguments of the main function. In the final implemented version of the CDS, we also used conformal prediction measures in the form of confidence and credibility of prediction metrics for each new sample presented to be evaluated by the model (Multimedia Appendix 1 [19-31]).

#### Clinical Recommendations

Given that the principal intended use of the CDS tool is to help health professionals plan customized psychological interventions for women at risk of poor mental health outcomes such as recovery from BC, an extensive set of personalized clinical recommendations was developed as follows: (1) potentially modifiable predictors of each of the key well-being outcomes that emerged from each of the 12 models included in the CDS tool were grouped into clinically meaningful intervention targets, (2) specific BOUNCE clinical recommendations were prepared for each set of intervention targets, and (3) health care professionals may choose to receive generic recommendations on how to address all substantial modifiable predictors that emerged from a given well-being prediction model or review predictor profile plots based on patient-level model-agnostic analyses and select specific intervention targets to receive appropriate treatment recommendations according to their expertise.

The BOUNCE clinical recommendations are not meant as any type of systematic psychological therapy for the patients but rather as a set of ideas that the professional can use to stimulate patients’ involvement in their treatment, empower them, and help them self-manage their experience with illness and its psychological sequelae in a way that is tailored to the needs of each patient. The BOUNCE clinical recommendations focus on patients’ resources to cope with, adjust to, and deal with the cancer experience and aim to foster their resilience.

## Results

### Overview

Of the total cohort of 706 women enrolled, 537 (76.1%) were followed up for 12 months, and 495 (70.1%) were followed up for 18 months. The average age of the women followed up for 12 and 18 months, respectively, was 55.5 (SD 8.2) years and 55.8 (SD 8.2) years. Most of the women followed up for 12 and 18 months had completed at least a high school education (498/537, 92.7% and 466/495, 94.1%, respectively), were married or in a relationship (406/537, 75.5% and 368/495, 74.3%, respectively), and were diagnosed with stage-I or stage-II BC (481/537, 89.6% and 446/495, 90.1%, respectively). The women followed up for 12 and 18 months were mostly treated with lumpectomy (397/537, 73.8% and 366/495, 73.9%, respectively), endocrine treatment (456/537, 84.9% and 418/495, 84.4%, respectively), radiotherapy (428/537, 79.7% and 395/495, 79.8%, respectively), or chemotherapy (280/537, 52.1% and 262/495, 52.9%, respectively).

### Classification Performance in Predicting 12-Month Mental Health Status (Models Ai-Aii)

Of the 537 women who were followed up for 12 months, 430 (80.1%) reported minimal or mild symptoms of anxiety or depression. The remaining 19.9% (107/537) of women comprised the group displaying an overall “poor” mental health outcome. Model A_i_ relied on the clinical, biological, and psychological measurements available at month 0 and month 3, whereas model A_ii_ relied on the clinical and biological measurements obtained at month 0 combined with the psychological measurements available at month 6. Correct prediction of poor outcomes was achieved for 78.9% (424/537) to 82% (440/537) of patients depending on the type and timing of the predictor measurements (Table 3). Moreover, the models identified the patients who exhibited good mental health status at month 12 with 76.9% (413/537) to 78.9% (424/537) certainty (area under the curve=0.81).

**Figure 1:**
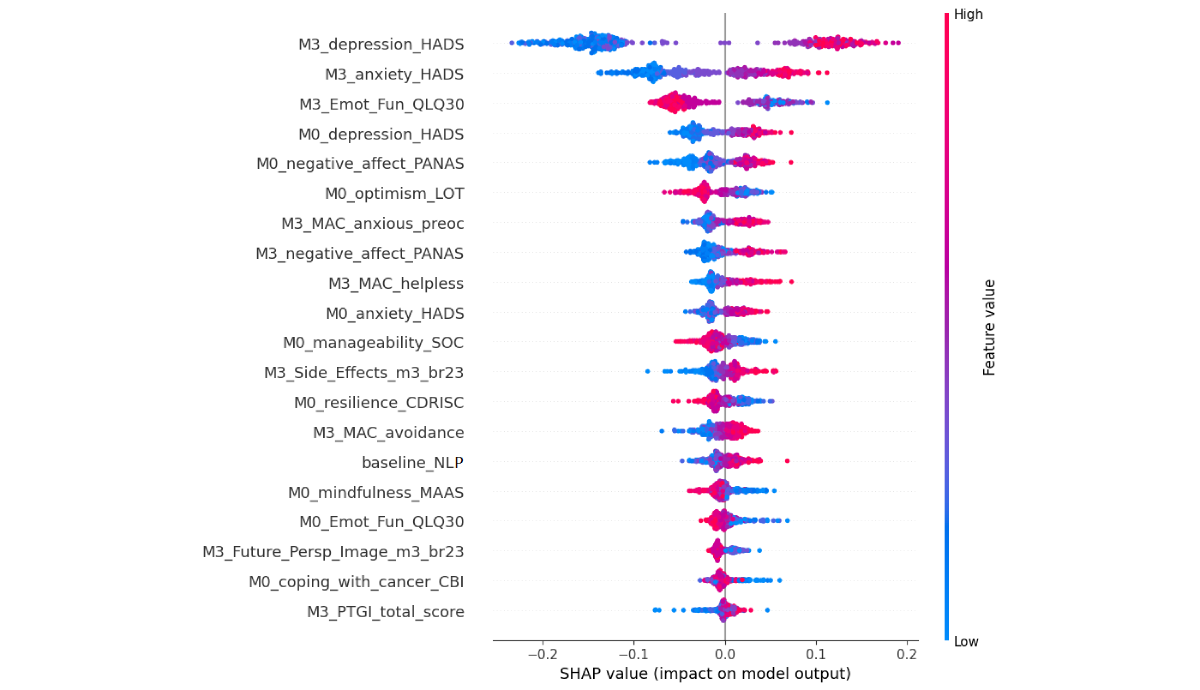
The selected features for model A_i_ ranked according to their relative importance (Shapley Additive Explanations [SHAP] values) for prediction of overall mental health status at month 12. "M0" indicates variables assessed at the time of enrollment in the study, and "M3" indicates variables assessed 3 months later. anxiety_HADS: average score on the anxiety subscale of the Hospital Anxiety and Depression Scale; br23: module of the European Organisation for Research and Treatment of Cancer Quality of Life questionnaire; CBI: Cancer Behavior Inventory; CDRISC: Connor-Davidson Resilience Scale; depression_HADS: average score on the depression subscale of the Hospital Anxiety and Depression Scale; Emot_Fun: emotional functioning; helpless: helplessness; LOT: Life Orientation Test; M0: month 0; M3: month 3; MAAS: Mindful Attention Awareness Scale; MAC: Mental Adjustment to Cancer scale; NLP: proportion of neutrophils in total leukocytes at M0; PANAS: Positive and Negative Affect Schedule; persp: perspective; preoc: preoccupation; PTGI: Posttraumatic Growth Inventory; QLQ30: Quality of Life Questionnaire–Cancer; SOC: Sense of Coherence–13 questionnaire.

As shown in Figure 1, important predictors of the 12-month decline in mental health status included variables measured at the time of enrollment in the study (before the onset of oncological treatments; month 0) as well as variables recorded at the 3-month follow-up (ie, during treatment; month 3). The highest-ranking features were depression severity at month 0 and month 3, anxiety symptom severity at month 3, and overall emotional functioning. Overall emotional state (negative affect) measured at baseline and month 3 and certain coping reactions at month 3 (ie, anxious preoccupation, avoidance, and helplessness) featured strongly among the most highly ranked predictors of mental health deterioration. Table S3 in Multimedia Appendix 1 [19-31] lists these variables. These factors could become the focus of systematic psychological interventions to enhance patients’ well-being and adaptation to BC. In addition, certain other variables emerged as substantial predictors of mental health, such as self-efficacy to cope with cancer (Cancer Behavior Inventory), resilience as a trait, and the ability to cope with trauma and optimism. Importantly, one of the biological variables measured at the time of diagnosis was highlighted as an important predictor (the proportion of neutrophils in total leukocytes).

**Table 3:** Model performance in predicting mental health status at 12 months after enrollment in the study.

Model type	Model group	Accuracy (%), mean (SD)	Sensitivity (%), mean (SD)	Specificity (%), mean (SD)	*F*_1_-score, mean (SD)	AUC^a^, mean (SD)
A	i	80 (4)	82 (10)	79 (4)	60 (6)	0.81 (0.5)
B	ii	78 (4)	79 (11)	77 (5)	57 (6)	0.78 (0.6)

^a^AUC: area under the curve.

### Personalized Risk Profiles for Poor Mental Health Through Month 12

**Figure 2:**
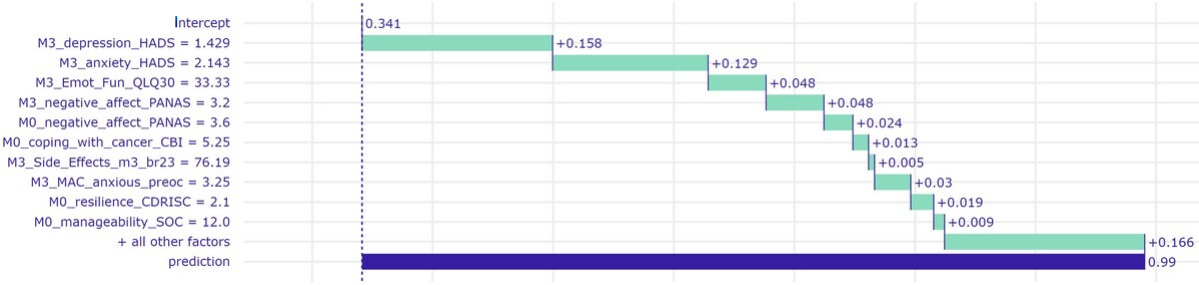
Breakdown profile of a patient who was correctly assigned to the poor mental health class at month 12 (high probability; 0.99). Prediction probability is shown on the horizontal axis (good mental health=0; poor mental health=1). This patient was given a high probability of belonging to the poor mental health class (0.99). A positive value assigned to a given score (green bars) indicates the degree of its contribution to a prediction of poor mental health. Actual patient scores on each predictor variable are shown on the left-hand side. Compared with the distribution of corresponding scores in the Predicting Effective Adaptation to Breast Cancer to Help Women to BOUNCE Back prospective study data set, this patient scored above the 75th percentile on month 3 (M3) depression and anxiety, negative affect (month 0 [M0] and M3), treatment side effects (M3), and anxious preoccupation (M3) and below the 25th percentile on emotional functioning, self-efficacy to cope with cancer, trait resilience (M0), and manageability (M0). anxiety_HADS: average score on the anxiety subscale from the Hospital Anxiety and Depression Scale; br23: module of the European Organisation for Research and Treatment of Cancer Quality of Life questionnaire; CBI: Cancer Behavior Inventory; CDRISC: Connor-Davidson Resilience Scale; depression_HADS: average score on the depression subscale from the Hospital Anxiety and Depression Scale; Emot_Fun: emotional functioning; MAC: Mental Adjustment to Cancer scale; PANAS: Positive and Negative Affect Schedule; preoc: preoccupation; QLQ30: Quality of Life Questionnaire–Cancer; SOC: Sense of Coherence–13 questionnaire.

**Figure 3:**
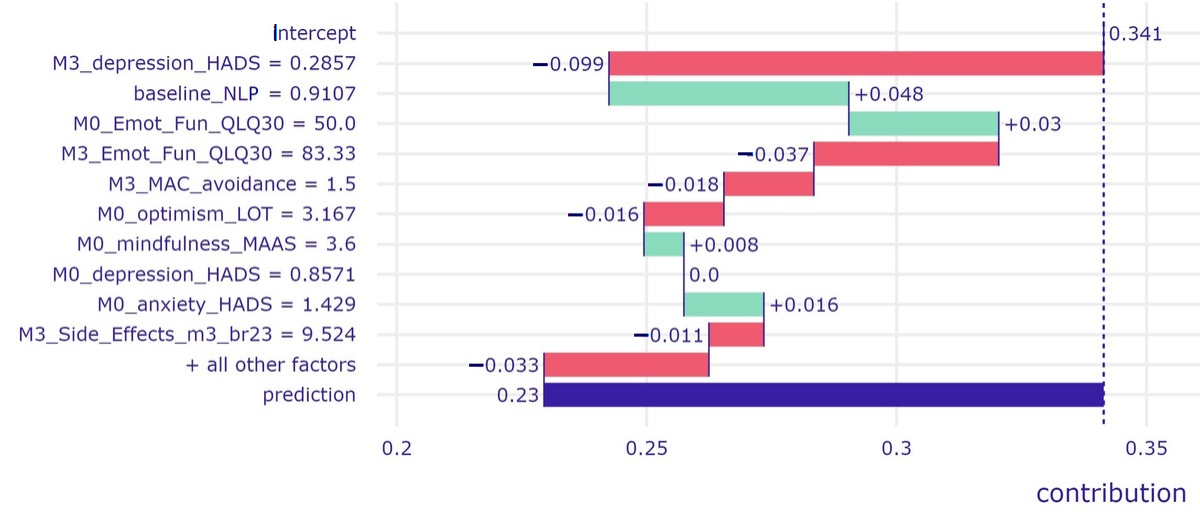
Breakdown profile of a patient who was correctly assigned to the good mental health status at month 12, as indicated by a low probability of belonging to the poor mental health class (0.23).

**Figure 4:**
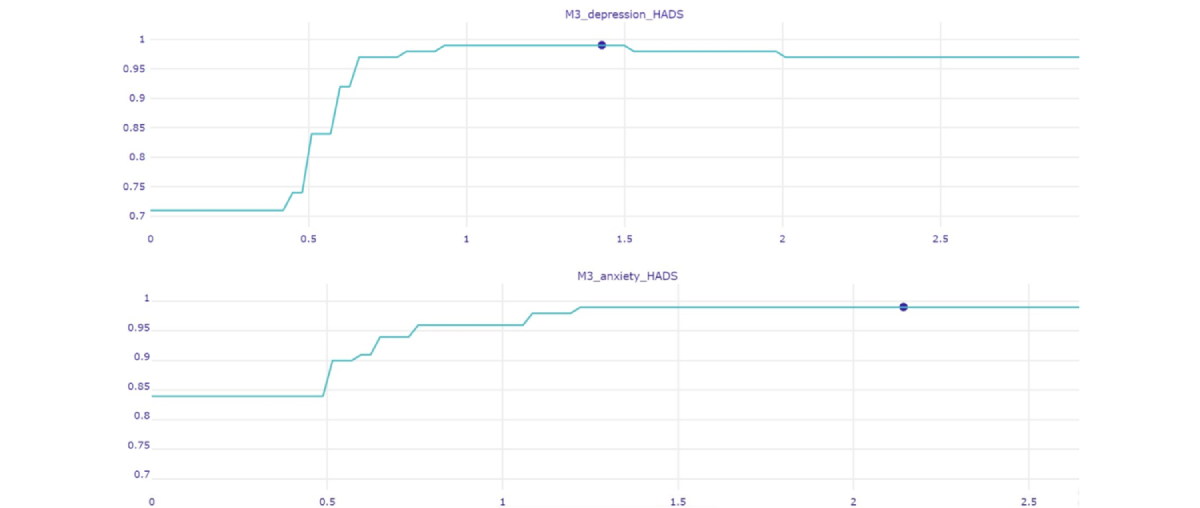
Patient-specific ceteris paribus plots for 2 of the highest-ranking features (severity of anxiety [lower panel] and depression [upper panel] symptoms at month 3 [M3]) of the patient shown in Figure 2, who was correctly assigned to the poor mental health class at month 12. Prediction probability is shown on the vertical axis (good mental health=0; poor mental health=1). The full range of possible scores on each predictor variable are plotted on the horizontal axis, whereas the patient’s actual reported score is indicated by a blue dot. anxiety_HADS: average score on the anxiety subscale from the Hospital Anxiety and Depression Scale; br23: module of the European Organisation for Research and Treatment of Cancer Quality of Life questionnaire; depression_HADS: average score on the depression subscale from the Hospital Anxiety and Depression Scale; Emot_Fun: emotional functioning; LOT: Life Orientation Test; MAAS: Mindful Attention Awareness Scale; M0: month 0; MAC: Mental Adjustment to Cancer scale; NLP: proportion of neutrophils in total leukocytes at M0; QLQ30: Quality of Life Questionnaire–Cancer.

**Figure 5:**
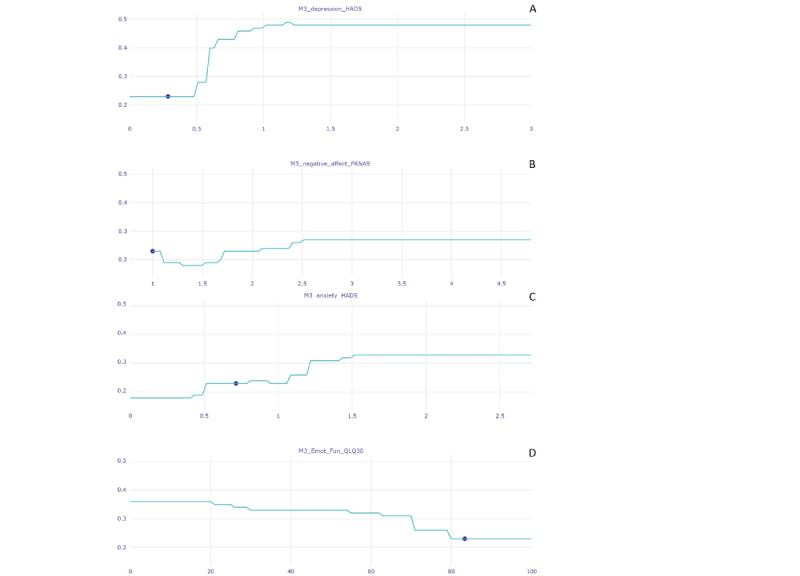
Patient-specific ceteris paribus plots for 4 of the highest-ranking features (severity of anxiety [C] and depression [A] symptoms, negative affect [B], and emotional functioning [D] at month 3 [M3]) of a patient who was correctly assigned to the good mental health class at month 12. Prediction probability is shown on the vertical axis (good mental health=0; poor mental health=1). The full range of possible scores on each predictor variable are plotted on the horizontal axis, whereas the patient’s actual reported score is indicated by a blue dot. anxiety_HADS: average score on the anxiety subscale from the Hospital Anxiety and Depression Scale; depression_HADS: average score on the depression subscale from the Hospital Anxiety and Depression Scale; Emot_Fun: emotional functioning; QLQ30: Quality of Life Questionnaire–Cancer.

Examples of breakdown profile plots for 2 randomly selected patients from each group are presented in Figures 2 and 3, whereas examples of ceteris paribus profiles for the same patients are presented in Figures 4 and 5. The estimated contribution of a subset of variables to a correct prediction of poor mental health is shown in Figure 2. Compared with the distribution of corresponding scores in the BOUNCE prospective study data set, this patient scored above the 75th percentile on month 3 depression and anxiety, negative affect (month 0 and month 3), treatment side effects (month 3), and anxious preoccupation (month 3) and below the 25th percentile on emotional functioning, self-efficacy to cope with cancer, trait resilience (month 0), and manageability (month 0). The 10 most highly ranked features selected by the BRF model for this specific instance-level prediction are displayed for demonstration purposes. These variables predominantly “facilitate” the adverse mental health outcome for this patient: relatively high scores on depression and anxiety; negative affect; side effects from treatment; and anxious preoccupation accompanied by relatively low levels of emotional functioning, self-efficacy to cope with cancer, sense of illness manageability, and trait resilience.

Figure 4 displays the ceteris paribus profiles for 2 key variables for the patient in Figure 2. The estimated response function of each variable was calculated while maintaining all other variables fixed. In this example, depression symptom severity scores (month 3) as low as 0.6 points (on a 0-3 scale) are sufficient to drive the prediction toward the poor mental health outcome at month 12 with great certainty (classification probability of >0.90) even if the values of all other predictor variables are held constant. A similar profile was observed for the severity of anxiety symptoms at month 3.

Figure 3 illustrates the breakdown profile of a patient who was correctly predicted to have displayed good mental health status over time (as indicated by very low probability for belonging to the poor mental health class [0.35]). Individual scores that *reduced* the probability of poor mental health (ie, contributed to a prediction of good mental health; red bars) included relatively low depression symptoms, avoidant coping, and treatment side effects, accompanied by relatively high emotional functioning and optimism. However, certain patient scores emerged as risk factors that could increase, albeit modestly, the probability of an adverse mental health outcome, namely, a relatively high proportion of neutrophils, low levels of overall emotional functioning, and mindfulness accompanied by relatively high levels of anxiety at baseline. The ceteris paribus plots (Figure 5) illustrate, among other points, how a small increase in depression symptom severity (from the observed 0.286 to 0.6 on a 0-3–point scale) could substantially increase the risk of a 12-month adverse mental health outcome for this patient. Similarly, the plots illustrate how a modest reduction in emotional functioning score (from the observed 83.33 to approximately 60 on a 0-100–point scale) could substantially increase the risk of poor mental health outcomes controlling for all other predictor variables.

### The Integrated CDS Tool

The CDS tool user has several options to accommodate specific clinical needs in terms of prediction end points and capabilities to engage in diverse prevention strategies, as well as according to the timing of available psychological and lifestyle data. These features are expected to facilitate the applicability of the CDS platform for a wider variety of clinical scenarios and settings.

The CDS tool was developed using a three-tier architecture: (1) the semantic tier, which includes all available data and serves as the basis for the analytical functionalities of the platform; (2) the AI tier, which consumes the available data and provides the main AI and ML modeling and execution capabilities of the platform; and (3) the application tier, which uses the services from the AI tier and presents the results of the models through the graphical user interface. The various layers communicate via well-defined representational state transfer application programming interfaces to implement the designed flow of information between them in a robust and solid manner.

**Figure 6:**
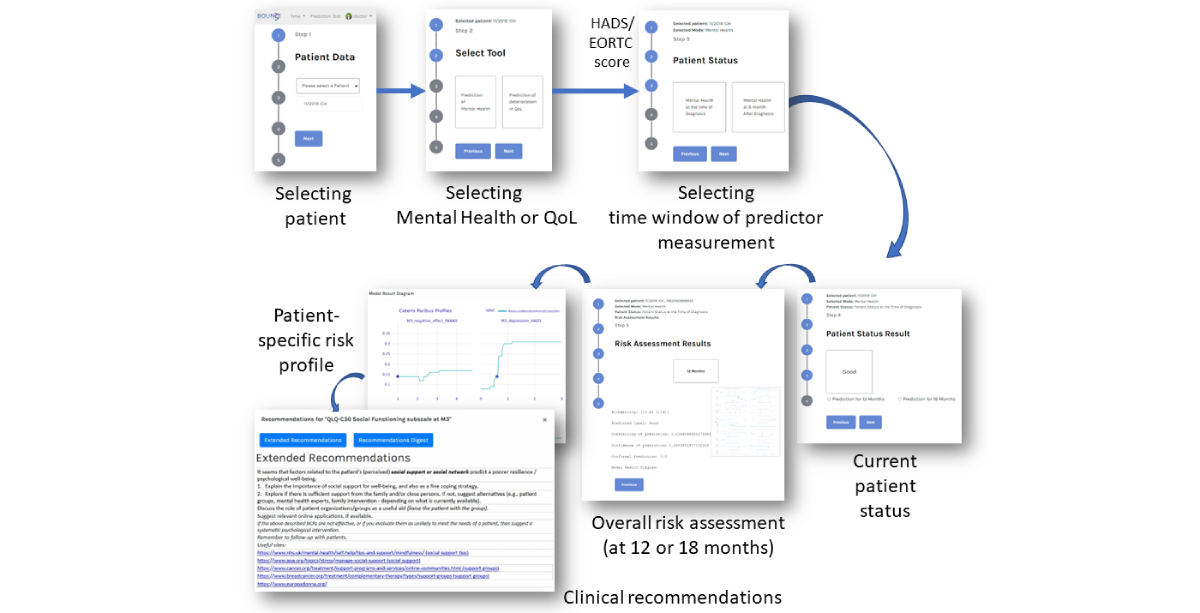
Workflow of the clinical decision support platform. EORTC: European Organisation for Research and Treatment of Cancer; HADS: Hospital Anxiety and Depression Scale; QoL: quality of life.

The CDS tool workflow is shown in Figure 6. Initially, the user selects to use either the “Prediction of Mental Health” tool (corresponding to model types A and B) or the “Prediction of deterioration in QoL” tool (model type C; step 2). If the former tool is selected, the patient’s total current Hospital Anxiety and Depression Scale score is requested; if the latter option is selected, their European Organisation for Research and Treatment of Cancer questionnaire score is requested. Next, the user selects the model group according to the available predictor data (ie, month 0 and month 3 data or month 0 and month 6 data; step 3). The patient’s *current* mental health (or, accordingly, overall QoL) category is shown (“poor” or “good”; step 4), and the user can then choose to estimate the predicted patient status at 12 or 18 months down the line. The selected models are executed, and the risk assessment results are visualized along with the probability, credibility, and confidence of the prediction (step 5).

If the predicted status of a given patient is *poor*, the CDS tool displays ceteris paribus plots allowing the user to compare the patient’s actual scores on each predictor variable with the recorded scores of the patients in the BOUNCE prospective clinical study. This step allows the user to form a tentative personalized risk and vulnerability profile for a given patient, potentially comprising relatively high scores on certain “risk” variables (such as anxious preoccupation) or relatively low scores on specific “protective” variables (such as coping strategies). The shape of the ceteris paribus plots further allows the user to identify patient characteristics that, if modified, could lead to a substantial change in the probability of the predicted adverse well-being outcome.

Finally, based on their clinical experience and specific clinical needs, users can select variables as targets for potential psychological interventions by clicking on their respective ceteris paribus plots. BOUNCE clinical recommendations are available in two forms: (1) an abbreviated version for clinicians who come in direct contact with the patient but are not trained in administering systematic psychological support and (2) an extended version for mental health professionals who have some training in psychological interventions. The BOUNCE clinical recommendations refer to a short list of basic cues and solutions that a health care professional can use to encourage the patient to more actively participate in their treatment and enhance their psychological health and QoL. The BOUNCE clinical recommendations are not meant as any type of systematic psychological therapy for the patients but rather as a set of ideas that the professional can use to stimulate patients’ involvement in their treatment, empower them, and help them self-manage their experience with illness and its psychological sequelae in a way that is tailored to the needs of each patient. The BOUNCE clinical recommendations focus on patients’ resources to cope with, adjust to, and deal with the cancer experience and aim to foster their resilience.

## Discussion

### Overview

The design of ML models included in the BOUNCE CDS tool was guided primarily by the potential future clinical utility of the forthcoming results. Thus, the supervised learning models included variables that can be readily available to practicing clinicians at major oncology centers in most countries, namely, medical, sociodemographic, and lifestyle variables integrated with a select set of psychosocial patient characteristics. In essence, the results of the BOUNCE predictive models presented in this paper are in full agreement with psychological theory and research on the factors involved in adaptation to severe illness, including BC [48,49]. However, the vast number of proposed predictors in the extant psycho-oncology literature prohibits their application in routine clinical practice. Thus, it is very difficult for knowledge-based approaches to the assessment of risk factors for adverse psychological outcomes to be widely adopted. In addition, these approaches are not intended to provide individualized profiles of risk factors that can be directly translated into customized clinical recommendations. To the best of our knowledge, the BOUNCE CDS tool is the first of its kind as it integrates ML-based prediction models that have been cross-validated in several ethnic and cultural settings with a data-driven method to identify potentially modifiable patient characteristics to improve psychological resilience during BC treatment.

Importantly, we used a rigorous analytic approach to mitigate some of the commonly observed pitfalls of ML approaches, namely, overfitting and poor model generalizability. Furthermore, we demonstrated their generalizability and interoperability by reducing their algorithmic bias while facilitating the interpretation of results according to the key challenges of XAI [50]. The guidelines for building trustworthy AI/ML systems with reliable components [51] were evaluated to ensure the trust and consistency of the models in the CDS tool. Seven requirements were considered for the implementation of trustworthy solutions in the platform: (1) transparency, (2) technical robustness and safety, (3) privacy and data governance, (4) diversity and fairness, (5) societal and environmental well-being, (6) accountability, and (7) human oversight throughout the models’ development and integration into the CDS tool [50]. Throughout the BOUNCE study and model development, close collaboration between modelers and clinicians ensured the quality and fidelity of the data sets (eg, whenever variable recoding and computation of relevant indexes were needed)*.* Clinician input was also instrumental in limiting the set of features entered into the ML models according to conventional psychometric criteria. In addition, robust cross-validation procedures were adopted to ensure that only the most relevant features were retained in the final prediction models. The behavior of these variables at a global level (both within and between classes) was inspected and confirmed to be relevant to the clinical question/prediction at hand. This approach is in line with the notion of transparency, that is, making the prediction process of clinically relevant models much more apparent and interpretable to clinicians and other stakeholders. These steps are important to conform to the rising demand for good AI/ML practices in real-world settings and guard against the potential liability of medical experts using AI-/ML-based solutions [51]. In this work, we took further steps to promote the effective deployment of the BOUNCE predictive models in health care settings in the context of XAI. Thus, ML model design afforded feature-specific visualizations at the global and local (patient) levels, which were incorporated into the CDS tool in the form of user-available and interpretable reports.

The BOUNCE approach encapsulates a number of *technological advances*. Chief among these is the development of ML classification models that provide input to a CDS system for predicting the psychosocial status of women with BC throughout the cancer continuum. This is built using predictive modeling technologies through a multidisciplinary approach involving social sciences and humanities, medicine, and computer science. To facilitate clinical utility, the CDS tool is supported by a dynamic electronic platform to allow for the input of targeted psychosocial, QoL, and lifestyle data by the patient and of medical data by the clinician. It should be noted that the analysis pipeline built into the CDS tool is comprehensive, considering a wide range of patient characteristics, and adaptable to the data that may actually be available to support a clinical decision for a given patient. Importantly, the tool can enhance clinician decision-making in terms of transparency and trust in XAI/ML models. Finally, the personalized risk assessment output is accompanied by 2 layers of clinical recommendations to accommodate both the specific needs of a given patient and the level or expertise and preference of the clinician.

The BOUNCE solution presented in this paper would not be possible without the extensive database of multimodal, multi-scale measurements obtained in a multicenter clinical study at 5 oncology centers with extensive experience in the comprehensive treatment of large numbers of patients with BC. The prospective study featured several measurement time points to consider multiple trajectories to clinical recovery and patient well-being (biological, social, environmental, lifestyle, occupational, and socioeconomic and psychosocial factors). Crucially, the study followed participants over a period of 18 months after the initial diagnostic procedures for BC. During this time, women are faced with several life-changing stressors (treatment decisions, side effects, and their impact on daily life). This period is crucial for the assessment of potential vulnerabilities that could predispose women to poor psychological well-being, as well as for providing expert support to promote behavioral and cognitive emotional adaptation strategies. In addition, the study measured multiple indexes of psychological resilience to ensure that all theoretically relevant aspects of this complex construct are considered and, ultimately, validated against objectively measurable clinical and psychosocial end points. The highest-ranking predictors of adverse mental health and QoL outcomes across the 12 models included in the CDS tool involved several common variables that can be classified into the following “clusters”: (1) negative affect, (2) coping with cancer responses and self-efficacy to cope with cancer, (3) a sense of control/positive expectations (ie, sense of coherence and optimism), (4) social and family support, (5) certain lifestyle factors (ie, exercise), and (6) certain treatment-related symptoms (eg, arm symptoms). These findings are consistent with the notion that adaptation to a severe health crisis is a complex process that is determined by (1) a variety of personal and interpersonal resources, such as expectations, lifestyle, or social support, which may buffer the negative impact of the situation and facilitate adaptation; (2) cognitive emotional processes, such as affect, emotion regulation, and self-efficacy to cope with cancer, which guide behaviors such as preoccupation and helplessness; and (3) contextual and specific stressor-related factors that may affect adaptation directly or indirectly, such as physical symptoms [52,53].

### Limitations

Data-related limitations should be considered in evaluating these results. The availability of a larger data set, possibly by oversampling patients in the smaller, “poor” outcome classes, may have enhanced the consistency of the results and possibly improved the overall prediction accuracy. We attempted to address this limitation by controlling for the ratio of predictors to cases. Moreover, the subsets of patients entered into each of the 12 alternative models were highly overlapping, ensuring the comparability of the model results. Importantly, the BOUNCE approach and model construction is flexible and adaptable to different cultural and clinical settings. Above all, the most direct method to validate these results and support the clinical utility of the BOUNCE CDS tool is through a prospective randomized controlled trial comparing personalized interventions informed by the tool’s clinical recommendations with a conventional intervention that does not consider individualized risk profiles.

The effort to build appropriate and valid clinical recommendations has 2 additional limitations. The first refers to the development of the BOUNCE clinical recommendations, and the second refers to their implementation. Specifically, the development of the BOUNCE clinical recommendations was based on the results of the prospective study that was conducted in 4 countries. Thus, the critical predictors to which the BOUNCE clinical recommendations correspond may vary for patients in different cultural settings. Carrying out similar research efforts in these countries as well may be an appropriate response to this limitation. In addition, the limited resources at many oncology centers may prevent the implementation of these clinical recommendations. However, it is noteworthy that the implementation of the BOUNCE clinical recommendations is not very demanding.

### Conclusions

Our results highlight the importance of early psychological responses to cancer diagnosis and related treatments and stress the necessity for timely intervention strategies to prevent substantial deterioration of patient well-being. The end product of the BOUNCE project paves the way for personalized risk assessment methods to identify patients at high risk of adverse well-being outcomes. This is a crucial step toward directing valuable resources to those patients with a more acute need for specialized psychological interventions. Finally, the BOUNCE approach highlights the usefulness of ML methods combined with carefully timed longitudinal measurements of a wide range of potential predictor variables. Such thoroughly cross-validated models can substantially reduce the length (and patient burden) of assessment batteries targeting complex psychological processes such as resilience and increase their feasibility in future research and clinical applications.

## Appendix

Multimedia Appendix 1 Additional information on measures, methodological details, and results.

## Abbreviations

AI: artificial intelligence
BC: breast cancer
BOUNCE: Predicting Effective Adaptation to Breast Cancer to Help Women to BOUNCE Back
BRF: balanced random forest
CDS: clinical decision support
HER2: human epidermal growth factor receptor 2
ML: machine learning
QLQ-C30: Quality of Life Questionnaire–Cancer
QoL: quality of life
XAI: explainable artificial intelligence

## Data Availability

The data that support the findings of this study can be made available upon reasonable request and with permission from the Predicting Effective Adaptation to Breast Cancer to Help Women to BOUNCE Back consortium (given that public availability of patient data was not covered by the study ethics approvals and corresponding patient consent forms).
