# Supplementary material for: Personalized Risk Analysis to Improve the Psychological Resilience of Women Undergoing Treatment for Breast Cancer: Development of a Machine Learning–Driven Clinical Decision Support Tool
Source: J Med Internet Res. 2023 Jun 12;25:e43838. doi: 10.2196/43838 (PMC10337304; doi:10.2196/43838)
Supplement: Multimedia Appendix 1 [file jmir_v25i1e43838_app1.docx]

**Personalized risk analysis to improve psychological resilience of women undergoing treatment for breast cancer: A Machine Learning-driven Clinical Decision Support Tool**

# Supplementary Material

^2^Georgios Manikis, ^2^Nicholas J. Simos, ^1,14^Konstantina Kourou, ^2^Haridimos Kondylakis, ^3^Paula Poikonen-Saksela, ^4,5^Ketti Mazzocco, ^6^Ruth Pat-Horenczyk, ^7,8^Berta Sousa, ^8,9^Albino J. Oliveira-Maia, ^3^Johanna Mattson, ^10^Ilan Roziner, ^4^Chiara Marzorati, ^2^Kostas Marias, ^11^Mikko Nuutinen, ^2,12^Evangelos Karademas and ^1,14^Dimitrios I. Fotiadis^*^

^1^Unit of Medical Technology and Intelligent Information Systems, Dept. of Materials Science and Engineering, University of Ioannina, Ioannina, Greece

^2^Foundation for Research and Technology-Hellas, Institute of Computer Science, Heraklion, Greece

^3^Helsinki University Hospital Comprehensive Cancer Center and University of Helsinki, Helsinki, Finland

^4^Applied Research Division for Cognitive and Psychological Science, European Institute of Oncology IRCCS, Milan, Italy

^5^Dept. of Oncology and Hemato-oncology, University of Milan, Milan, Italy

^6^School of Social Work and Social Welfare,The Hebrew University of Jerusalem, Israel

^7^Breast Unit, Champalimaud Clinical Centre, Champalimaud Foundation, Lisbon, Portugal

^8^Champalimaud Research and Clinical Centre, Champalimaud Foundation, Lisbon, Portugal

^9^NOVA Medical School, NMS, Universidade Nova de Lisboa, Lisbon, Portugal

^10^Dept. of Communication Disorders, Sackler Faculty of Medicine, Tel Aviv University, Israel

^11^Nordic Healthcare Group, Helsinki, Finland

^12^Dept. of Psychology, University of Crete, Rethymno, Greece

^13^ School of Medicine, University of Crete, Heraklion, Greece

^14^Foundation for Research and Technology-Hellas, Biomedical Research Institute, Ioannina, Greece

**indicates corresponding author (*[*fotiadis@uoi.gr*](mailto:fotiadis@uoi.gr)*)*

*Authors GM and NJS contributed equally to this work.*

# Measures

***Positive and Negative affect****.* The Positive and Negative Affectivity Schedule (PANAS) [20] was used to evaluate positive (10 adjectives; Cronbach’s $\alpha=0.84$) and negative affect (10 adjectives; Cronbach’s $\alpha=0.75$). A 5-point Likert type scale was adopted to assess affect over the past week. Higher scores represent higher levels of positive and negative affect, respectively.

***Fear of Cancer Recurrence Inventory****.* The 9-item Fear of Cancer Recurrence Inventory (FCRI) questionnaire was used to measure the fear of a recurrence event [21]. Each item of the questionnaire is rated on a Likert type scale ranging from 0 (“not at all” or “never”) to 4 (“a great deal” or “all the time”). The total score can be obtained by summing the responses to all items. Higher scores indicate higher levels of FCR.

***Health Status.*** To evaluate patients’ global health status, the BR-23 module of the European Organization for Research and Treatment of Cancer (EORTC) QLQ questionnaire was used [19]. This module comprises 23 questions related to the (i) disease symptoms, (ii) side effects of treatment (surgery, chemotherapy, radiotherapy and hormonal treatment), (iii) body image, (iv) sexual functioning and (v) future perspective. It should be noted that a linear transformation was applied to the raw scores to reach a range from 0 to 100. Also, patients’ functioning and well-being were assessed with the function (i.e., physical, role, emotional, cognitive, and social functioning) and symptoms scales (e.g., fatigue, pain) of the European Organization for Research and Treatment of Cancer (EORTC) QLQ-C30 questionnaire [19].

***Illness perception and coping responses.*** The brief version of the Cancer Behavior Inventory (CBI-B) measure [22] was used to assess a general sense of perceived self-efficacy to cope with the illness-related difficulties and needs. A single score measure of coping self-efficacy was yielded (Cronbach’s $\alpha=0.89$) with higher scores indicating higher confidence in coping with illness. The Mental Adjustment to Cancer scale (MAC) [23] was used to estimate patients' coping responses to cancer. The scale includes five reliable dimensions: (i) fighting spirit, (ii) helplessness, (iii) anxious preoccupation, and (iv) avoidance. A 4-point Likert type scale indicate the coping responses of BC patients. Also, the Perceived Ability to Cope with Trauma (PACT) questionnaire was used to estimate the flexibility in coping across different potentially traumatic events [24]. Two scales are measured related to: (i) the focus on processing the trauma (trauma focus), and (ii) the focus on moving beyond the trauma (forward focus). An overall PACT flexibility score was created to evaluate both types of coping. Finally, to assess any potential positive responses to the entire stressful experience, we used the total score on the 14-Post-Traumatic Growth Inventory (PTGI short form) questionnaire (with higher scores indicating better posttraumatic growth) [25].

***Social support and family resilience.*** The modified Medical Outcomes Study Social Support Survey (mMOS-SS) was used to assess social support, which has been shown to provide many benefits related to overall health and well-being [26]. It consists of 8 items and the total score was calculated by summing all response values (Cronbach’s $\alpha=0.92$). Higher total and subscale mMOS-SS scores reflect stronger social support. For the assessment of family resilience the Walsh Family Resilience Questionnaire [27] was used. For the purposes of the BOUNCE study, two subscales were used: (i) communication and cohesion and (ii) perceived family coping. A higher total score indicates higher levels of family resilience.

***Resilience as a personality characteristic (trait).*** The Connor-Davidson Resilience Scale was used to assess resilience as a trait (CD-RISC) [28]. The scale includes 10 items for quantifying the level of self-perceived resilience (e.g. ability to adapt to change; achieving my goals). Each item is rated on a 5-point Likert type scale from 0 (“not true at all”) to 4 (“true nearly all the time”) with higher total scores reflecting higher resilience levels (Cronbach’s $\alpha=0.89$).

***Emotion regulation and relevant strategies.*** The Cognitive Emotion Regulation Questionnaire (CERQ – short) was used to identify the cognitive emotion regulation strategies (or cognitive coping strategies) that BC patients followed when experiencing negative events or situations [29]. A 5-item Likert type scale was used for each item ranging from 1 (“(almost) never”) to 5 (“(almost) always”). In addition, the Mindful Attention Awareness Scale (MAAS) [30] was used to assess the patients’ characteristic of mindfulness. A total score is considered by summing all patients’ responses with higher scores reflecting higher levels of dispositional mindfulness.

***Other personality characteristics.*** Sense of coherence was assessed based on the Sense of Coherence (SOC)-13 questionnaire (Cronbach’s $\alpha=0.81$, for the total score). Comprehensibility (5 items), manageability (4 items), and meaningfulness (4 items) were measured on a 7-point (Likert-type) response scale (from 1 (lower) to 7 (higher)) with higher total scores indicating higher level of sense of coherence. Generalized optimism was assessed with the Life Orientation Test (LOT)–Revised (Cronbach’s $\alpha=0.71$) [31].

**Analyses**

*Model uncertainty*

In the final implemented version of the CDS, we have also utilized conformal prediction measures in the form of confidence and credibility of prediction metrics, presented for each new sample presented to be evaluated by the model (see Supplementary Material). Conformal prediction estimates are error bound on a per-instance basis without specifying prior probabilities. As a useful indicator of the model’s confidence in a specific new prediction, this result is presented to the user in the final version of the CDS after the input of a new patient’s data. The implementation used for the calculation of confidence and credibility metrics is the “nonconformist” package: <https://github.com/donlnz/nonconformist> (<http://donlnz.github.io/nonconformist/_autosummary/nonconformist.icp.IcpClassifier.html#nonconformist.icp.IcpClassifier>*).*

# Results

# *Classification Performance for prediction of 12-month decline in mental health status or QoL*

Table S1 lists model classification performance for predicting mental health change (models B_i-ii_), and quality of life change (models C_i-ii_) at 12 months post-baseline. Classification results for Models A_i-ii_ (described in detail in the main text) are also included for comparison.

# *Prediction of decline in mental health (Type B)*

Women who reported minimal or mild symptoms of anxiety or depression at baseline and followed to M12 were eligible for this model (n=378). Of those 328 maintained low HADS scores throughout the first year post-baseline (Stable-Good Mental Health group), while the remaining 50 patients had clinically significant symptomatology at M12 (Deteriorated Mental Health group). As shown in Table S1, sensitivity and specificity values ranged between 75-84% and 83-86%, respectively, depending on the type and timing of predictor measurements.

# *Prediction of decline in QoL (Type C)*

Women who reported fair global quality of life at baseline (in relation to their peers) and followed to M12 were eligible for this model (n=398). Of those 314 (78.9%) maintained fair global QoL throughout the first year post-baseline (Stable-Good QoL group); the Deteriorated QoL group (n=92) comprised women who scored in the lower 25^th^ percentile on the EORTC scale at M12. As shown in Table S1, sensitivity and specificity values ranged between 74-76% and 80-82%, respectively, depending on the type and timing of predictor measurements.

**Table S1.** Model performance in predicting overall mental health (A), mental health deterioration (B) or overall quality of life deterioration (C) at 12 months post-baseline (mean ± SD).

| **Model Type** | **Model Group** | **Accuracy** | **Sensitivity** | **Specificity** | **F1** | **AUC** |
| --- | --- | --- | --- | --- | --- | --- |
| **A** | i | 80 ± 4 | 82 ± 10 | 79 ± 4 | 60 ± 6 | 81 ± 5 |
|  | ii | 78 ± 4 | 79 ± 11 | 77 ± 5 | 57 ± 6 | 78 ± 6 |
| **B** | i | 79 ± 4 | 84 ± 12 | 86 ± 5 | 54 ± 8 | 86 ± 6 |
|  | ii | 82 ± 4 | 75 ± 14 | 83 ± 5 | 53 ± 8 | 79 ± 6 |
| **C** | i | 79 ± 5 | 74 ± 13 | 80 ± 6 | 58 ± 9 | 77 ± 7 |
|  | ii | 81 ± 5 | 76 ± 13 | 82 ± 6 | 61 ± 8 | 79 ± 6 |

# *Classification Performance in predicting 18-month outcomes*

Table S2 lists model classification performance for predicting overall mental health status (models A_i-ii_) mental health decline (models B_i-ii_), and quality of life change (models C_i-ii_) at 18 months post-baseline.

# *Models predicting overall mental health (Type A)*

All 495 women who were followed up to 18 months were considered in these analyses. The majority (n=418; 84.4%) reported minimal or mild symptoms of anxiety or depression at the 18-month follow up (as indicated by a HADS score<8 points). The remaining 77 (15.6%) scored ≥8 points. As shown in Table S2, sensitivity and specificity values ranged between 72-78% and 76-79%, respectively, depending on the type and timing of predictor measurements.

# *Models predicting decline in mental health (Type B)*

Women who reported minimal or mild symptoms of anxiety or depression at baseline and were followed to M18 were eligible for this model (n=363). Of those 327 maintained low HADS scores throughout the first year post-baseline (Stable-Good Mental Health group), while the remaining 36 patients had clinically significant symptomatology at M18 (Deteriorated Mental Health group). As shown in Table S2, sensitivity and specificity values ranged between 70-81% and 75-83 %, respectively, depending on the type and timing of predictor measurements.

# *Models predicting decline in QoL (Type C)*

Women who reported fair global quality at baseline (in relation to their peers) and followed to M18 were eligible for this model (n=321). Of those 295 maintained fair global QoL throughout the first year post-baseline (Stable-Good QoL group); the Deteriorated QoL group (n=26) comprised women who scored in the lower 25^th^ percentile on the EORTC scale at M18. As shown in Table S2, sensitivity and specificity values ranged between 71-80% and 82-84%, respectively, depending on the type and timing of predictor measurements.

**Table S2.** Model performance in predicting overall mental health (A), mental health deterioration (B) or overall QoL deterioration (C) at 18 months post-baseline.

| **Model Type** | **Model Group** | Accuracy | Sensitivity | Specificity | F1 | AUC |
| --- | --- | --- | --- | --- | --- | --- |
| **A** | i | 76 ± 4 | 72 ± 11 | 76 ± 5 | 49 ± 6 | 74 ± 5 |
|  | ii | 79 ± 4 | 78 ± 11 | 79 ± 5 | 54 ± 6 | 79 ± 5 |
| **B** | i | 74 ± 5 | 70 ± 16 | 75 ± 6 | 43 ± 9 | 73 ± 8 |
|  | ii | 83 ± 4 | 81 ± 14 | 83 ± 5 | 57 ± 9 | 82 ± 7 |
| **C** | i | 79 ± 6 | 71 ± 13 | 82 ± 7 | 62 ± 9 | 76 ± 7 |
|  | ii | 83 ± 6 | 80 ± 12 | 84 ± 7 | 68 ± 9 | 82 ± 6 |

**Table S3a**. Selected final features per model Type and model Group, Month 12 predictions

| **Type A, group i** | **Type A, group ii** | **Type B, group i** | **Type B, group ii** | **Type C, group i** | **Type C, group ii** |
| --- | --- | --- | --- | --- | --- |
| M3 Depression | M6 Depression | M3 Negative affect | M6 PTSD symptoms | M3 Global QoL | M6 Global QoL |
| M3 Anxiety | M6 Coping w/ cancer | M3 Depression | M6 Depression | M3 Role Function | M6 Physical Function |
| M3 Negative affect | M6 PTSD symptoms | M3 Emotional Function | M6 Anxiety | M3 Physical Function | M6 Fatigue |
| M3 Emotional Function | M6 Negative affect | M3 Anxiety | M6 Coping w/ cancer | M3 Depression | M6 Coping w/ cancer |
| M3 Anxious Preoccupation | M6 Anxiety | M0 Manageability | M6 Emotional Function | M3 Social Function | M6 Illness Consequences |
| M0 Anxiety | M6 Illness Emotional Representations | M0 Depression | M6 Illness Emotional Representations | M3 Side Effects | M6 Depression |
| M0 Negative affect | M6 Emotional Function | M3 Anxious Preoccupation | M6 Negative affect | M3 _Fatigue | M6 Role Function |
| M0 Depression | M6 Global QoL | M3 Helplessness | M6 Illness Consequences | M0 Coping w/ cancer | M6 Positive Affect |
| M3 Helplessness | M6 Fear of recurrence | M0 Negative affect | M6 Global QoL | M3 Emotional Function | M6 Fear of recurrence |
| M0 Optimism | Neutrophil/ Leucocyte ratio | M0 Coping w/ cancer | M6 _Fatigue | Sick leave | M6 Side Effects |
| M0 Trait Resilience | M6 Illness Consequences | M0 Optimism | M6 Fear of recurrence | M0 Mindfulness | M6 Social Function |
| M0 Manageability | M6 Side Effects | M0 Anxiety | Neutrophil/Leucocyte ratio | M3 Body Image | M6 Negative affect |
| M0 Coping w/ cancer | M6 Physical Function | Neutrophil/Leucocyte ratio | M6 Side Effects | M3 Positive Affect | M6 Emotional Function |
| M3 Avoidance | M6 Positive Affect | Thrombocytes | M6 Social Function | M0 Manageability | M6 Anxiety |
| Neutrophil/Leucocyte ratio | M6 _Fatigue | M3 Avoidance | Thrombocytes | M3 Family coping | Thrombocytes |
| M0 Mindfulness | M6 Illness cycling | M3 _Fatigue | M6 Illness timeline | M3 Anxious Preoccupation | M6 PTSD symptoms |
| M0 Emotional Function | M6 Social Function | M3 Social support | M6 Positive Affect | M0 Emotional Function | M6 Future Image |
| M3 PTGI total | M6 Illness timeline | M0 Trait Resilience | M6 Illness cycling | M3 Community Cohesion | Neutrophil/ Leucocyte ratio |
| M3 Side Effects | Thrombocytes | M3 Global QoL | M6 Physical Function | M0 Anxiety | age |
| M3 Future Image | Creatinine | M0 Meaningfulness | M6 Illness Coherence | Neutrophil/ Leucocyte ratio | Sick leave |

**Table S3b.** Selected final features per model Type and model Group, Month 18 predictions

| **Type A, group i** | **Type A, group ii** | **Type B, group i** | **Type B, group ii** | **Type C, group i** | **Type C, group ii** |
| --- | --- | --- | --- | --- | --- |
| M3 Depression | M6 Depression | M3 Emotional Function | M6 Depression | M3 Global QoL | M6 Global QoL |
| M0 Manageability | M6 Emotional Function | M3 Depression | M6 Coping w/ cancer | M3 Side Effects | M6 Depression |
| M3 Anxiety | M6 Anxiety | M0 Manageability | M6 Anxiety | M3 Depression | M6 Illness Consequences |
| M3 Anxious Preoccupation | M6 Illness Emotional Representations | M3 Negative affect | M6 Negative affect | M3 _Fatigue | M6 Side Effects |
| M0 Anxiety | M6 PTSD symptoms | M3 Anxious Preoccupation | M6 Illness Emotional Representations | M3 Anxious Preoccupation | M6 Role Function |
| M0 Optimism | M6 Coping w/ cancer | M3 Anxiety | M6 PTSD symptoms | M3 Emotional Function | M6 Physical Function |
| M3 Negative affect | M6 Negative affect | M3 Global QoL | M6 Emotional Function | M3 Role Function | M6 _Fatigue |
| M3 Emotional Function | M6 Illness Consequences | Neutrophil/ Leucocyte ratio | M6 _Fatigue | M3 Anxiety | M6 PTSD symptoms |
| M0 Depression | M6 Fear of recurrence | M0 Coping w/ cancer | M6 Global QoL | M3 Physical Function | M6 Positive Affect |
| M3 Avoidance | Neutrophil/ Leucocyte ratio | M0 Depression | M6 Social Function | M3 Social Function | M6 Coping w/ cancer |
| M0 Coping w/ cancer | M6 Side Effects | M0 Optimism | M6 Illness Consequences | M0 Manageability | M6 Anxiety |
| M3 Helplessness | M6 Illness cycling | M0 Anxiety | Neutrophil/ Leucocyte ratio | Sick leave | M6 Future Image3 |
| Neutrophil/ Leucocyte ratio | M6 Positive Affect | M3 Helplessness | M6 Side Effects | M3 Cognitive Function | M6 Fear of recurrence |
| M3 Community Cohesion |  |  | M6 Fear of recurrence | M3 Helplessness | M6 Social Function |
|  | Thrombocytes | Sick leave | M6 Cognitive Function | M3 Arm Symptoms | Neutrophil/ Leucocyte ratio |
| Sick leave | M6 Global QoL | M3 Avoidance | M6 Positive Affect | Thrombocytes | M6 Emotional Function |
| M0 Mindfulness | M6 Physical Function | M0 Mindfulness | Thrombocytes | M0 Coping w/ cancer | Creatinine |
| M3 Social support | M6 _Fatigue | M0 Comprehe-nsibility | M6 Arm Symptoms | Creatinine | Thrombocytes |
| M0 Compre-hensibility | M6 Illness Coherence | Thrombocytes | M6 Illness cycling | M3 Negative affect | M6 Illness Emotional Representations |
| M3 Family coping | M6 Illness timeline | M0_exercise | M6 Illness timeline | M3 Positive Affect | Sick leave |

**Table S4:** Sample sizes per class for month 12 prediction models (corresponding to Table S1 results)

| **Model Type** | **Model Group** | Class ‘0’ | Class ‘1’ |
| --- | --- | --- | --- |
| **A** | i | 397 | 92 |
|  | ii | 375 | 85 |
| **B** | i | 308 | 48 |
|  | ii | 296 | 43 |
| **C** | i | 207 | 50 |
|  | ii | 200 | 49 |

**Table S5:** Sample sizes per class for month 18 prediction models (corresponding to Table S2 results)

| **Model Type** | **Model Group** | Class ‘0’ | Class ‘1’ |
| --- | --- | --- | --- |
| **A** | i | 397 | 72 |
|  | ii | 379 | 68 |
| **B** | i | 256 | 41 |
|  | ii | 250 | 39 |
| **C** | i | 179 | 56 |
|  | ii | 173 | 52 |

Multimedia Appendix: The BOUNCE platform can be accessed online at <https://bounce.ics.forth.gr/>. Access is regulated with username/password and interested readers can communicate with the project coordinator to obtain a demo username/password.
